# Cardiopulmonary function after robotic exoskeleton-assisted over-ground walking training of a patient with an incomplete spinal cord injury: Case report

**DOI:** 10.1097/MD.0000000000018286

**Published:** 2019-12-16

**Authors:** Yun-Chol Jang, Hyeng-Kyu Park, Jae-Young Han, In Sung Choi, Min-Keun Song

**Affiliations:** Department of Physical & Rehabilitation Medicine, Regional CardioCerebroVascular Center, Chonnam National University Medical School & Hospital, Gwangju City, Republic of Korea.

**Keywords:** cardiopulmonary function, exoskeleton, gait training, incomplete spinal cord injury

## Abstract

**Rationale:**

Spinal cord injury (SCI) patients who experience difficulties with independent walking use gait-assistive devices such as a cane, walker, or wheelchair. Few studies have explored gait patterns or cardiopulmonary function in chronic SCI patients after powered exoskeleton training. We investigated whether the cardiopulmonary function of a patient with an incomplete chronic cervical SCI and a hemiplegic gait pattern could be improved by walking training using a powered exoskeleton (Angelegs).

**Patient concerns:**

A 57-year-old male was diagnosed with an SCI at C3-C4. The right upper and lower limb motor functions differed when evaluated before entry into the program. Motor function was good in the right leg but poor in the left one. Before program entry, the patient could walk for about 10 m using a cane. He did not have a history of severe medical or psychological problems and was not cognitively impaired.

**Diagnosis:**

The patient was tetraplegia with incomplete SCI at C3-C4.

**Interventions:**

The patient was trained for 6 weeks using a powered exoskeleton. The training program consisted of sit-to-stand and stand-to-sit movements, maintenance of balanced standing for 5 minutes, and walking for 15 minutes.

**Outcomes:**

After 6 weeks of training, gait speed improved in the timed up-and-go test, and cardiac function was enhanced as measured by the metabolic equivalent and VO_2_ tests.

**Lessions:**

Walking training using a powered exoskeleton can facilitate the effective rehabilitation and improve the gait speed and cardiopulmonary function of patients with chronic SCIs or strokes.

## 1 Introduction

Spinal cord injuries (SCIs) account for 180,000 injuries annually worldwide (26 per million subjects).^[1]^ SCIs are associated with various symptoms depending on the damaged spinal level; these include motor weaknesses of the upper and lower extremities, sensory abnormalities, muscular atrophy, pain, spasticity, and contracture.^[2,3]^ The risks of cardiovascular disease, respiratory problems, and osteoporosis increase when SCI patients find it difficult to walk independently.^[3–5]^ SCI patients who experience difficulties in independent walking use gait-assistive devices, such as a cane, a walker, or a wheelchair. Recently, various powered robotic exoskeletons have been used for mobility and gait training. According to Asselin et al (2013) and Esquenazi et al (2016), SCI patients trained using a lower-limb powered exoskeleton improved in terms of walking distance and speed, and their spasticity was reduced.^[6,7]^ Although several studies on powered exoskeletons have been recently published, few have explored gait patterns or cardiopulmonary function in patients with chronic SCIs after powered exoskeleton training. Also, few studies have examined patients with hemiplegic gait patterns. Here, we report changes in the gait pattern and cardiopulmonary function of a patient with a chronic incomplete SCI and hemiplegic features who was trained using a lower-limb powered exoskeleton, the Angelegs.

## 2 Methods

### 2.1 Ethical approval

All training and evaluation were conducted at Chonnam National University Hospital Rehabilitation Center. The study was approved by the Institutional Review Board of Chonnam National University Hospital (approval no. CNUH-2018-020). The patient was well informed about the study and voluntarily agreed to participate. Informed consent was obtained from the patient before the study.

### 2.2 Robotic exoskeleton

The Angelegs is composed of hip, knee, and ankle segments, of which only the hip and knee joints are motorized. A rechargeable battery is carried on the lower back. The battery allows up to 5 hours of use with a full charge. The total weight is about 10 kg, and each motor can instantaneously generate a continuous assistive force of 24.3 Nm. Pneumatic sensors are located in both insoles. The pneumatic sensors are weighted and calibrated while the patient stands with the Angelegs. When the patient starts to walk, the pneumatic sensors in the shoes can detect the pressure on the toe and heel and analyze the gait phase. This sensor sends the ground force data to the actuators in the hip and knee joints to provide proper assistive force adjusted to the gait phase. The actuators in the Angelegs’ hip and knee joints have kinematic sensors that detect the joint angle. These sensors analyze the angular data at the hip and knee joints during the gait phase. All sensors are programmed to deliver the correct assistive force to the hip and knee joints while the patient is moving. In this process, the Angelegs can provide the force based on the patient's gait phase and muscle strength of both legs. The Angelegs can support 4 types of movement modes: sit-to-stand, stand-to-sit, standing, and walking.

## 3 Case report

A 57-year-old male who had been diagnosed with an SCI at C3-C4 3 years before was admitted to the Chonnam National University Hospital Rehabilitation Center to participate in our program and was trained for 6 weeks. The right upper and lower limb motor functions differed when evaluated before entry into the program. Lower right leg motor function was good, but lower left leg function was poor. Before program entry, the patient could walk for about 10 m using a cane. He lacked severe medical and psychological problems and was not cognitively impaired.

**Figure 1:**
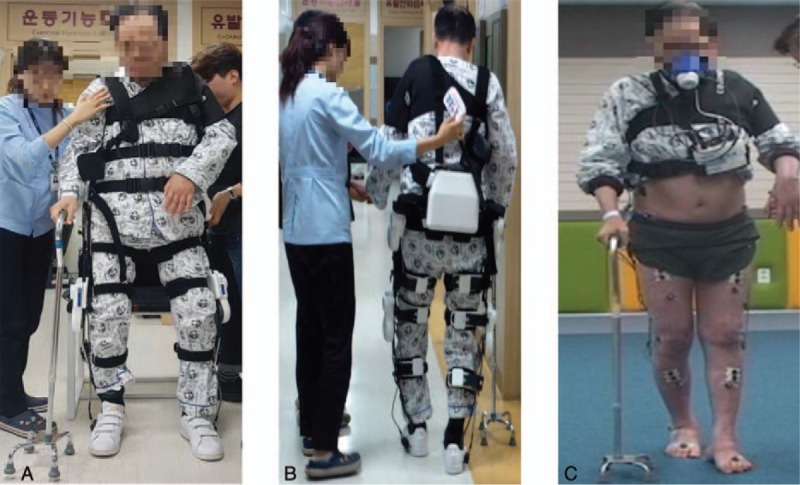
Robotic exoskeleton-assisted overground walking training. (A), (B) Gait training wearing Angelegs; (C) 3D gait analysis wearing portable gas analyzer.

The Angelegs Training program was scheduled for five 30-minute sessions per week for 6 weeks, for a total 30 sessions. Before program entry, he underwent three 30-minute pre-training sessions on the Angelegs. Training was conducted in the rehabilitation center of Chonnam National University Hospital; a single physical therapist supervised all training. The training program consisted of sit-to-stand and stand-to-sit movements, maintenance of standing balance for 5 minutes at a time, and walking for 15 minutes. He used a cane to maintain balance during training. Walking proceeded on flat ground for 10 m at a gait speed that was the best that the patient could attain (Fig. 1). During the program, the patient received the physical and occupational therapies (30-minute sessions) he had previously been prescribed.

A physical therapist evaluated functional outcomes using range of motion (ROM) tests; the Manual Muscle Test (MMT); the Modified Ashworth Scale (MAS); the Korean-Modified Barthel Index (K-MBI); Functional Ambulatory Category (FAC) scoring; 3-dimensional dynamic posturography (PRO-KIN system, TecnoBody Srl, Dalmine BG, Italy); EuroQol-5D (EQ-5D) scoring; a 10-m walking test; a timed up-and-go test, and 3-dimensional gait analysis before (T0) and after training (T1). The patient walked 10 m a total of 10 times in the flat aisle and walked normally as during the gait analysis. In addition, the patient wore a portable gas analyzer (model K4B2, COSMED Srl, Rome, Italy) that measured the VO_2_ levels and the metabolic equivalent (MET).

The patient visited our rehabilitation clinic 6 weeks after the end of the program (T2) and underwent all tests again. There were no adverse events during these assessments.

The 10-m walk test (at a comfortable pace) time decreased from 89.00 s (T0) to 86.50 s (T1) and 86.47 s (T2). In the up-and-go test, the speed increased from 6.8 cm/s (T0) to 9.7 cm/s (T1) and 10.3 cm/s (T2) (Table 1). However, the gait pattern did not differ at T0, T1, and T2.

**Table 1:**
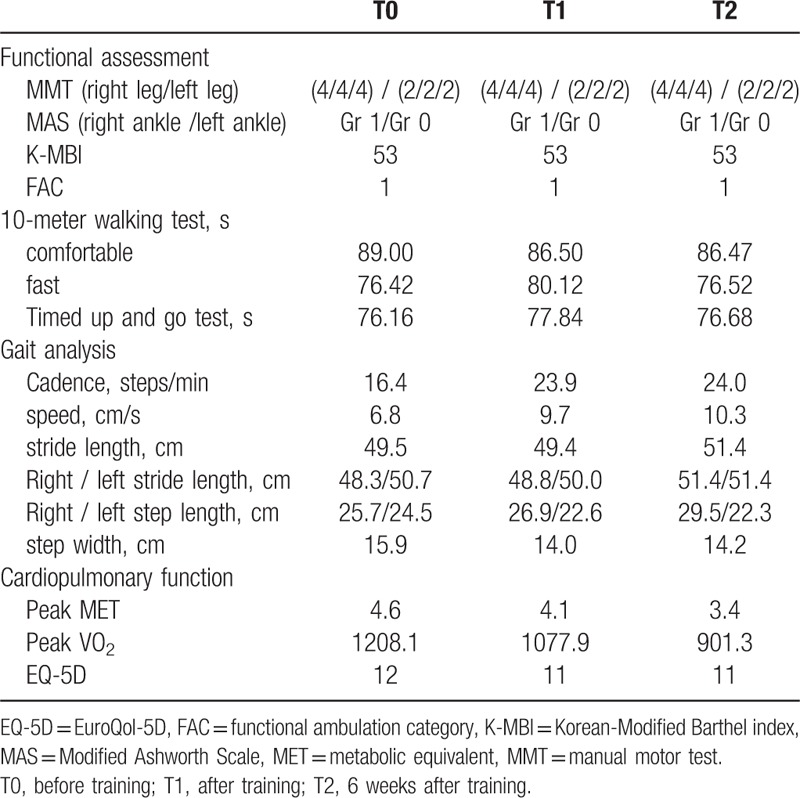
Changes of gait, physical, and cardiopulmonary function.

MET and VO_2_ were measured to evaluate the patient's cardiopulmonary function. MET decreased from 4.6 (T0) to 4.1 (T1) and 3.4 (T2). VO_2_ decreased from 1208.1 mL (T0) to 1077.1 mL (T1) and 901.3 mL (T2). However, other physical functions showed no improvement, such as ROM, MMT, MAS, K-MBI, FAC, stride length, step width, or EQ-5D data change over time (Table 1).

## 4 Discussion

We performed several functional assessments in addition to cardiopulmonary function, such as ROM, muscle strength, muscle tone, activities of daily living, balance and gait function, and quality of life. However, not all assessments showed improvement after training. These results are similar to those of previous studies on chronic SCIs.^[1,6,8]^ This suggests that training with a robotic exoskeleton may not facilitate the neurogenesis necessary to achieve significant functional gain in patients with chronic SCI. However, Louie et al (2015) showed that walking speed was significantly associated with injury level during training using an exoskeleton.^[8,9]^ Walking speed was faster when the injury level was lower. Our patient exhibited improved gait speed after training, despite injury at the upper cervical level (C3), which is typically associated with poor gait speed. The patient's peak VO_2_ and MET decreased at T1 (after completion of training), and the improvements were maintained at T2 (6 weeks later); the same activity thus required less effort. Cardiopulmonary function improved after training using a powered exoskeleton; the improvement was maintained for 6 weeks after training.

Many previous studies have shown that powered exoskeletons provide total external support to patients with complete SCI during gait training.^[1]^ However, patients with incomplete SCIs have their own muscle strength. When these patients are training using a robotic exoskeleton, full external support can be an impediment to training because it can limit the patient's voluntary walking efforts and weaken the patient's muscle strength. Therefore, it is important to help patients with incomplete SCIs use their muscles appropriately during gait training using a robotic exoskeleton. In this case report, the robotic exoskeleton provided the proper force according to the gait phase and the patient was able to strengthen his own muscles. In addition, the patient's energy consumption and cardiorespiratory function were improved during this robotic exoskeleton training program.

We found that robotic exoskeleton training improved the gait speed and cardiopulmonary function of a patient with an incomplete SCI. This patient was a paraplegic; the muscle strength of the right leg was good and that of the left leg was poor. This pattern is similar to that of patients with hemiplegia caused by stroke or Brown-Sequard syndrome. This case report provides evidence that patients with a chronic incomplete paraplegic pattern as well as the chronic hemiplegic pattern can improve clinical function after training using a powered exoskeleton. However, this is only a single case report and it cannot predict the outcome of all SCI patients. Therefore, further studies on robotic exoskeleton training for incomplete SCI patients are needed.

## Author contributions

**Investigation:** Yun-Chol Jang, Min-Keun Song.

**Resources:** Jae-Young Han, In Sung Choi, Min-Keun Song.

**Supervision:** Hyeng-Kyu Park, Jae-Young Han, In Sung Choi.

**Writing – original draft:** Yun-Chol Jang.

**Writing – review & editing:** Min-Keun Song.

Min-Keun Song orcid: 0000-0001-8186-5345.
